# Comparison of protein expression between formalin-fixed core-cut biopsies and surgical excision specimens using a novel multiplex approach

**DOI:** 10.1007/s10549-019-05163-6

**Published:** 2019-02-22

**Authors:** Mariana Ferreira Leal, Ben P. Haynes, Fiona A. MacNeill, Andrew Dodson, Mitch Dowsett

**Affiliations:** 1 Ralph Lauren Centre for Breast Cancer Research, Royal Marsden Hospital, The Royal Marsden NHS Foundation Trust, 4th Floor Wallace Wing, 203 Fulham Road, London, SW3 6JJ UK; 2 Breast Cancer Now Research Centre, The Institute of Cancer Research, Fulham Road, London, SW3 6JB UK; 3 Breast Unit, The Royal Marsden NHS Foundation Trust, Fulham Road, London, SW3 6JJ UK

**Keywords:** Breast cancer, Immunohistochemistry, NanoString technology, Multiplex protein analysis, Phosphorylation, Tissue fixation

## Abstract

**Purpose:**

We evaluated whether multiplex protein quantification using antibody bar-coding with photocleavable oligonucleotides (NanoString) can be applied to evaluate protein expression in breast cancer FFPE specimens. We also assessed whether diagnostic core-cuts fixed immediately at time of procedures and surgical excision sections from routinely fixed breast cancers are affected by the same fixation related differences noted using immunohistochemistry (IHC).

**Methods:**

The expression of 26 proteins was analysed using NanoString technology in 16 pairs of FFPE breast cancer core-cuts and surgical excisions. The measurements yielded were compared with those by IHC on Ki67, PgR and HER2 biomarkers and pAKT and pERK1/2 phosphorylated proteins.

**Results:**

When considered irrespective of sample type, expression measured by the two methods was strongly correlated for all markers (*p* < 0.001; *ρ* = 0.69–0.88). When core-cuts and excisions were evaluated separately, the correlations between NanoString and IHC were weaker but significant except for pAKT in excisions. Surgical excisions showed lower levels of 8/12 phosphoproteins and higher levels of 4/13 non-phosphorylated proteins in comparison to core-cuts (*p* < 0.01). Reduced p4EBP1, pAMPKa, pRPS6 and pRAF1 immunogenicity in excisions was correlated with tumour size and mastectomy specimens showed lower p4EBP1 and pRPS6 expression than lumpectomy (*p* < 0.05).

**Conclusions:**

Our study supports the validity of the new multiplex approach to protein analysis but indicates that, as with IHC, caution is necessary for the analysis in excisions particularly of phosphoproteins. The specimen type, tumour size and surgery type may lead to biases in the quantitative analysis of many proteins of biologic and clinical interest in excision specimens.

**Electronic supplementary material:**

The online version of this article (10.1007/s10549-019-05163-6) contains supplementary material, which is available to authorized users.

## Introduction

Proteins are currently the most suitable target for anti-cancer drugs and protein biomarkers may help in therapy stratification. Immunohistochemistry (IHC) for both predictive and prognostic tests based on protein biomarkers has been applied to guide oncologists in their use of targeted therapies for breast cancer patients [1–3]. However, standard IHC is still generally considered a semi-quantitative approach to evaluate protein expression. Moreover, accurate IHC analysis is highly dependent on the methodology, observer and the quality of the tissue [4, 5].

It is well known that visual scoring is inherently subjective and both intra-observer and inter-observer variability have been commonly reported. This variability slows the progress of biomarker discovery and delivery of precision medicine [6]. Moreover, the most common staining methods (such as the “brown staining” with diaminobenzidine, DAB) have a limited dynamic range and different batches can present variations in the staining intensity in standard IHC. Immunofluorescent (IF) staining has improved the quantification of proteins [7] and digital pathology can increase the reliability and speed of analysis. The degree of agreement among manual and the automated methods (‘Man versus Machine’ comparison) has been published for the scoring of several breast cancer biomarkers, including HER2, ER, PgR and Ki67 [6, 8–10]. However, digital image analysis even in combination with fluorescence staining is still mainly used for the analysis of a single or a few biomarkers per slide and both technologies are limited in their availability [11].

Recently, a new method of protein quantification has become available with a number of advantages. This uses antibody bar-coding with photocleavable DNA oligonucelotides allowing multiplexed quantitative protein measurements and system-wide profiling on small amounts of tissue extracts [12]. After oligonucleotide cleavage from antibodies, a hybridization process is done and the oligonucleotide tags are quantified using the NanoString nCounter^®^ Analysis System (NanoString Technologies, Seattle, WA, USA) as commonly performed for gene expression analysis. Initial epitope retrieval and primary antibody binding steps are still required as per standard IHC. Therefore, this methodology is likely to be affected in a similar way to IHC by pre-analytical factors, such as cold ischemic time, intraoperative hypoxia, section thickness, type and duration of fixation and processor protocols [13].

We previously undertook a systematic evaluation of the semi-quantitative IHC expression of ER, PgR, HER2, Ki67 and the phosphorylated proteins pAKT and pERK1/2 in core-cut and excision specimens from primary breast cancer since clinical management involves assessment of both type of samples and particularly in clinical trials, a comparison between the two [14]. An extreme loss of staining of pAKT and pERK1/2 phosphoproteins was observed in excision samples compared with core-cut biopsies, changes that are likely related to suboptimal fixation [14]. Our previous study called attention to these changes leading to possible bias in clinical research and provided key evidence to form the basis of future guidelines for companion diagnostic tests [13].

Here, we evaluated whether the novel NanoString method of protein quantification using oligonucleotide bar-coded antibodies can be applied as an alternative methodology to evaluate protein expression in breast cancer formalin-fixed paraffin-embedded (FFPE) specimens. In particular, using a wide range of antibodies available, we assessed whether diagnostic core-cuts fixed immediately at time of procedures and surgical excision sections from routinely fixed primary breast cancers are affected by the same fixation related differences we noted by IHC [14]. This investigation has special relevance for “window of opportunity” studies where data from core-cuts are commonly compared with data from excision specimens from the same patients.

## Materials and methods

### Sample collection

We accessed tissues collected from a previous study given their optimal suitability for answering the central questions [14]. Core-cut biopsies (14-gauge needle) and surgical excision FFPE specimens were both available from 16 patients with ER positive primary breast cancer. Samples were from patients with a median age of 59 years [interquartile range (IQR) = 11; 51–62]. Median tumour size was 33.5 mm (IQR = 25; 20–45) and 7 patients (43%) were node-positive.

Core-cut biopsies were taken immediately after tumour resection and placed in neutral-buffered formalin. The surgical excision specimens were also placed in neutral-buffered formalin and subjected to the histopathology department’s routine fixation for breast tumours: lumpectomy samples (50% of the cases) were left unsliced until the next morning, and mastectomy samples (50%) were sliced at intervals of about 10 mm to allow penetration of formalin. Ethical approval was provided by the Royal Marsden Hospital (RMH) and all patients gave written informed consent.

### Protein expression analysis by IHC

The IHC expression of the non-phophorylated proteins Ki67, PgR and HER2 and phosphorylated proteins pAKT and pERK1/2 by IHC was available from our previous study [14]. Briefly, PgR (clone 16), pAKT (Ser473) and pERK1/2 (Thr202/204) were assessed by H-score. Ki67 (clone MIB-1) expression was measured by counting the numbers of positive and negative staining invasive tumour cells in 10 high-power fields (10 HPF’s) to derive a percentage of positive invasive cells staining with any intensity. HER2 was categorized as 0, 1+, 2+ or 3+ as per HercepTest™ (Agilent Dako, UK) and the American Society of Clinical Oncology/College of American Pathologists (ASCO/CAP) guidelines [15].

### Protein expression analysis by NanoString technologies

One 5-µm section from each FFPE block was cut on to a slide for protein analysis. A 4-µm section from each was also stained with haematoxylin and eosin (H&E) and used to identify areas with ≥ 40% invasive tumour cells. Non-invasive tissue areas were needle dissected away from invasive disease in the 5-µm sections before incubation of antibody mix. The expression of 26 target proteins and 3 controls (positive control: Histone H3; negative controls: anti-rabbit and anti-mouse IgG antibodies) were measured using the nCounter^®^ Vantage 3D™ Protein Solid Tumor Panel for FFPE (NanoString Technologies, Seattle, WA, USA). Commercial protocols from NanoString for hybridization and detection were followed with minor modifications as follows. After deparaffinization and rehydration, epitope retrieval was performed with low pH citrate buffer in a Pre-Treatment-link (Agilent Dako, UK) and samples were incubated overnight with the antibody mix at 4 °C. To cleave tags from antibodies, slides were exposed to UV for 3 min in UV Stratalinker 1800 (Strategene, USA).The dilution of nCounter^®^ oligonucleotide tags from the bound antibodies was optimized based on the analysis of 12 samples (6 core-cuts; 6 surgical excisions) run with 3 different dilutions. In all cases, the results of the 3 dilutions on a single sample clustered together on hierarchical clustering (Supplemental Fig. 1). Based on these results, cleaved tags were diluted 10x prior to denaturation and hybridization with reporter and capture probes (NanoString Technologies) since this maximized the detection of makers with no significant impact on background levels. Protein lysates were denatured at 95 °C for 5 min before hybridization with TagSet master mix at 65 °C for 20 h.

Raw counts from the nCounter^®^ FLEX Analysis System (NanoString Technologies) were normalized by the geometric mean of the counts from the 6 internal ERCC (External RNA Controls Consortium) positive controls to take into account the efficiency of the hybridization. Background correction was done by subtracting the geometric mean of the 6 ERCC negative control probes and then the 2 non-specific IgG controls. To adjust for differences in sample input, data were normalized to the level of Histone H3, which was confirmed to present the lowest variation across the studied samples. Expression values were log2 transformed for statistical analysis. For the direct comparison with IHC data, signals from IgG controls were not subtracted.

Zeros values were set as half of lowest expression detected for the respective protein.

### Statistical analysis

Descriptive data are presented as median and interquartile range (IQR). The correlation between protein expression by NanoString and IHC was evaluated by Spearman correlation test. Spearman correlation was also carried out to investigate the correlation between the proteins. Wilcoxon signed-rank test was used to compare the protein expression between cores and surgical excisions. Mann–Whitney U test was used to investigate whether protein expression varies according the type of surgery and to compare protein expression between clusters of samples. P values were two-sided and all confidence intervals were at the 95% level.

## Results

### Protein expression analysis by NanoString technologies is highly correlated with standard IHC

**Fig. 1:**
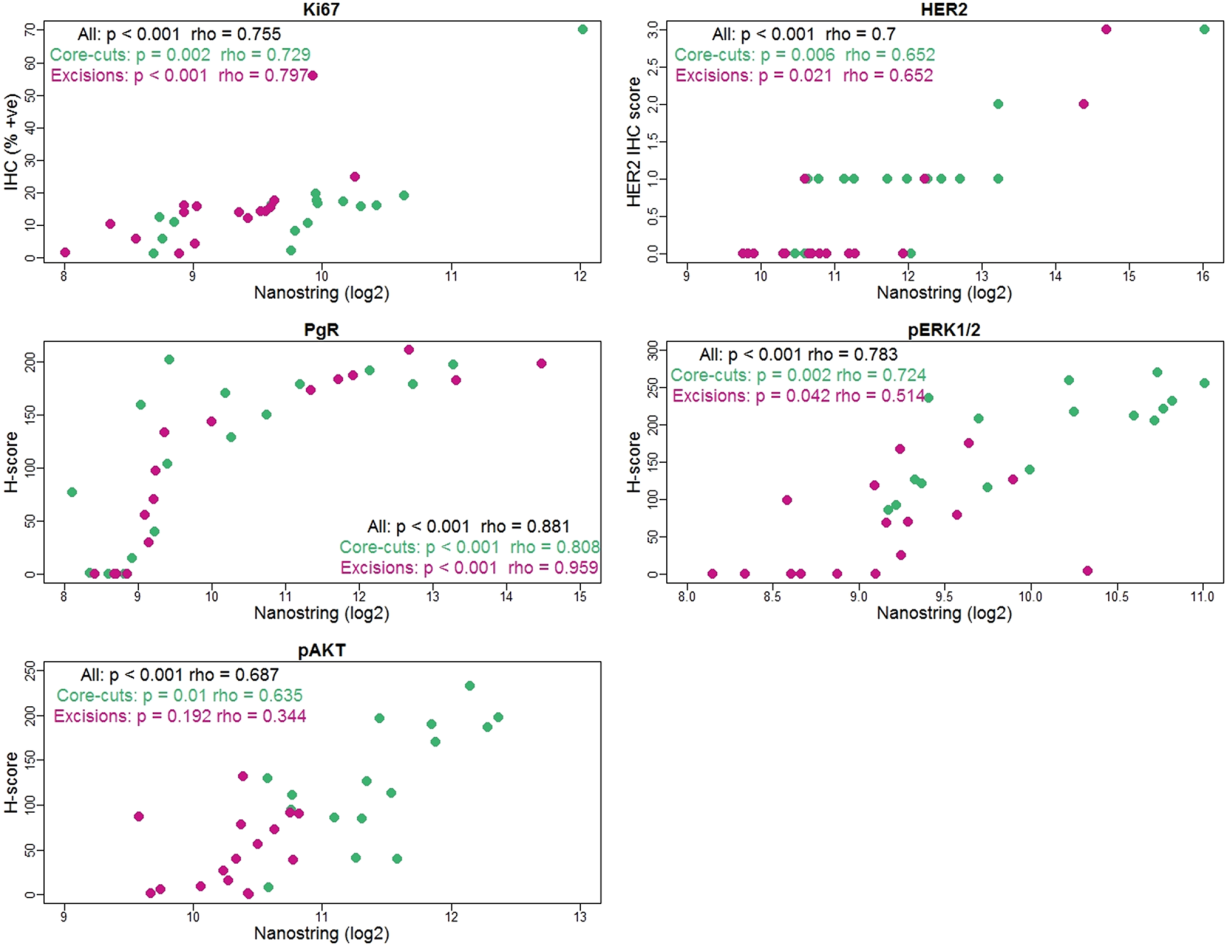
Correlation between protein expression measured by NanoString and IHC. Green dots: core-cut biopsies; Dark pink: excision samples. Coefficient of correlation (*ρ*) and *p*-value by Spearman correlation test are shown. PgR antibody recognizes different regions in NanoString (isoform B) and IHC (isoform A and B as routinely applied in clinical practice)

We initially assessed the correlation between the biomarker measurements by NanoString and IHC. When considered irrespective of sample type, the expression of both phosphorylated and non-phosphorylated proteins measured by both methods was significantly strong correlated (*ρ* = 0.69–0.88, *p* < 0.001 for all comparisons; Fig. 1). However, when core-cuts and surgical excisions were evaluated separately, the correlations between NanoString and IHC were weaker but still apparent except for pAKT measurements in excisions (Fig. 1).

### Differences in the phosphoprotein expression between core-cuts and excision specimens

**Fig. 2:**
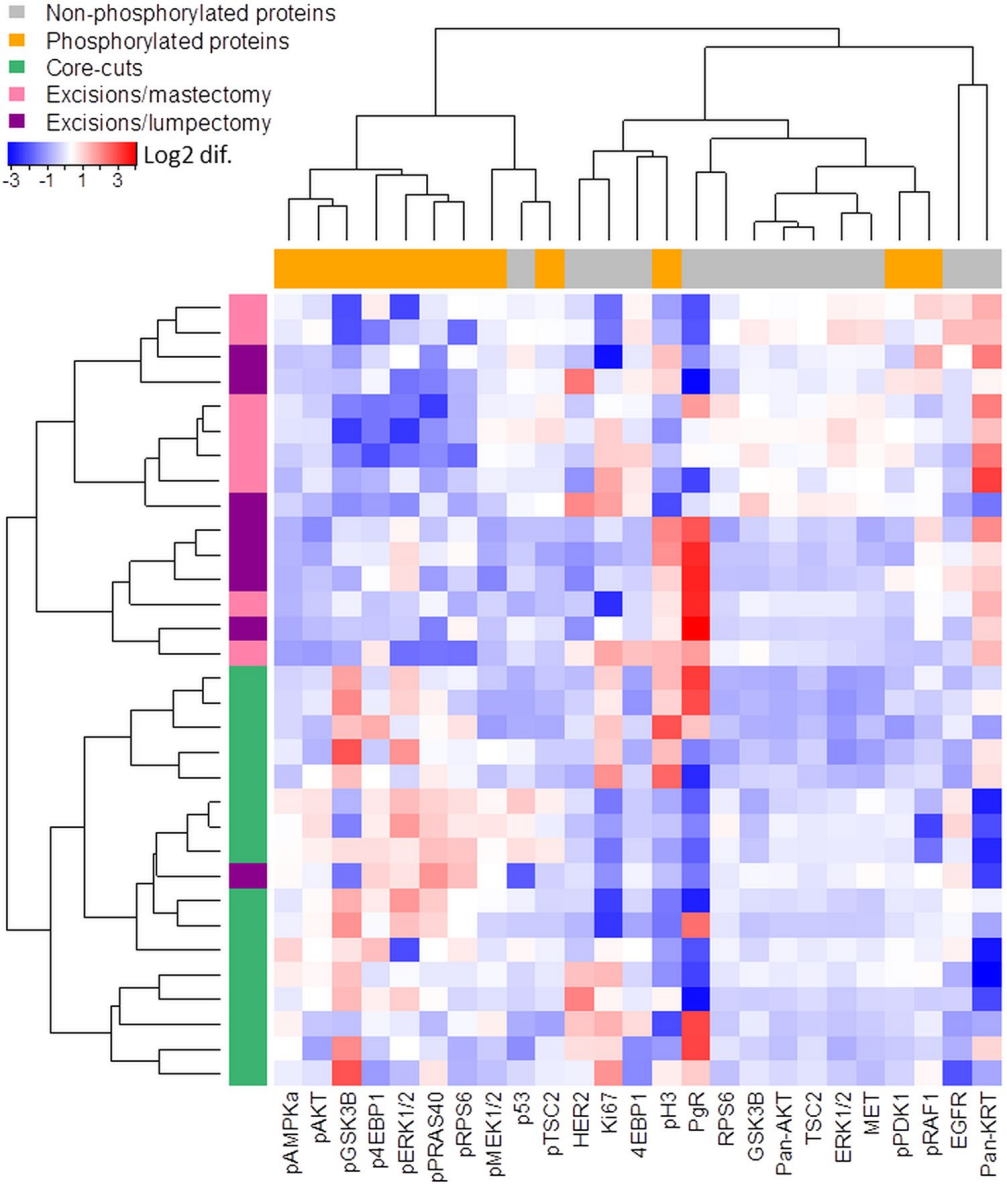
Heatmap based on Spearman Correlation test. Columns values were scaled by log2 difference in relation to the mean of each protein. Grey: non-phosphorylated proteins; orange: phosphorylated proteins; Green: core-cut biopsies; Pink: excisions/mastectomy; Purple: excisions/lumpectomy

Among the 26 studied proteins, only pEGFR was not detected in both core-cut and excision specimens and was therefore excluded from the following analysis. Using the expression data of the 25 remaining proteins, the studied samples were classified by unsupervised 2-way hierarchical clustering which yielded 2 main clusters in each direction: for sample type, one cluster was composed by excision specimens and another cluster by all core-cuts samples and one excision samples; and for protein type, one cluster was composed largely of phosphoproteins and one largely by non-phosphorylated proteins (Fig. 2).

**Fig. 3:**
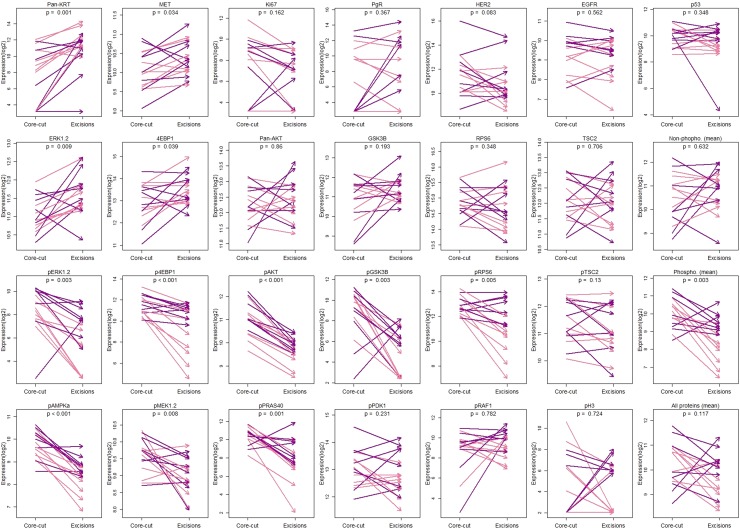
Comparison between cores and surgical excisions specimens. pEGFR was not detected above background in the studied samples and it is not shown. Pink: mastectomy. Purple: lumpectomy. p-value of Wilcoxon signed-rank test is shown. Zero values were set as half of the lowest expression detected of the respective protein

As previously reported using IHC and in agreement with the correlation analysis above described, Ki67, PgR and HER2 expression did not differ significantly between cores and excisions (*p* > 0.05; Figs. 2, 3) while pERK1/2 (14/16 with > 50% reduction; *p* = 0.003) and pAKT (mean reduction = 59.5%; *p* < 0.001) showed markedly lower levels in the excision samples (Fig. 3). There was also a trend for HER2 to be reduced in surgical excisions (*p* = 0.086) with 8/16 samples showing more than 50% of reduction in relation to matched core-cut biopsies.

In addition to pERK1/2 and pAKT, 6 other phosphorylated proteins also had significantly lower levels in surgical excisions in comparison to paired core-cuts (Fig. 3): p4EBP1 (mean reduction = 54.2%; *p* < 0.001), pGSK3B (14/16 with > 75% reduction; *p* = 0.003), pRPS6 (mean reduction = 40%; *p* = 0.005), pAMPKa (mean reduction = 55.1%; *p* < 0.001), pMEK1/2 (mean reduction = 26.3%; *p* = 0.008) and pPRAS40 (mean reduction = 34.1%; *p* = 0.001). Four phosphorylated proteins (pTSC2, pPDK1, pRAF1 and pH3) did not show a significant difference in expression between the paired core and excision samples.

### Differences in the non-phosphorylated proteins expression between core-cuts and excision specimens

Of the 13 non-phosphorylated proteins measured, 4 had significantly increased levels in surgical excisions compared to core-cuts (Fig. 3): pan-KRT (13/16 samples with > 100% increase; *p* = 0.001), 4EBP1 (mean increase = 87%; *p* = 0.039), ERK1/2 (mean increase = 69.5%; *p* = 0.009) and MET (mean increase = 27.9%; *p* = 0.034) and none had significantly decreased levels.

### Difference in the protein levels with the type of surgery and tumour size

**Fig. 4:**
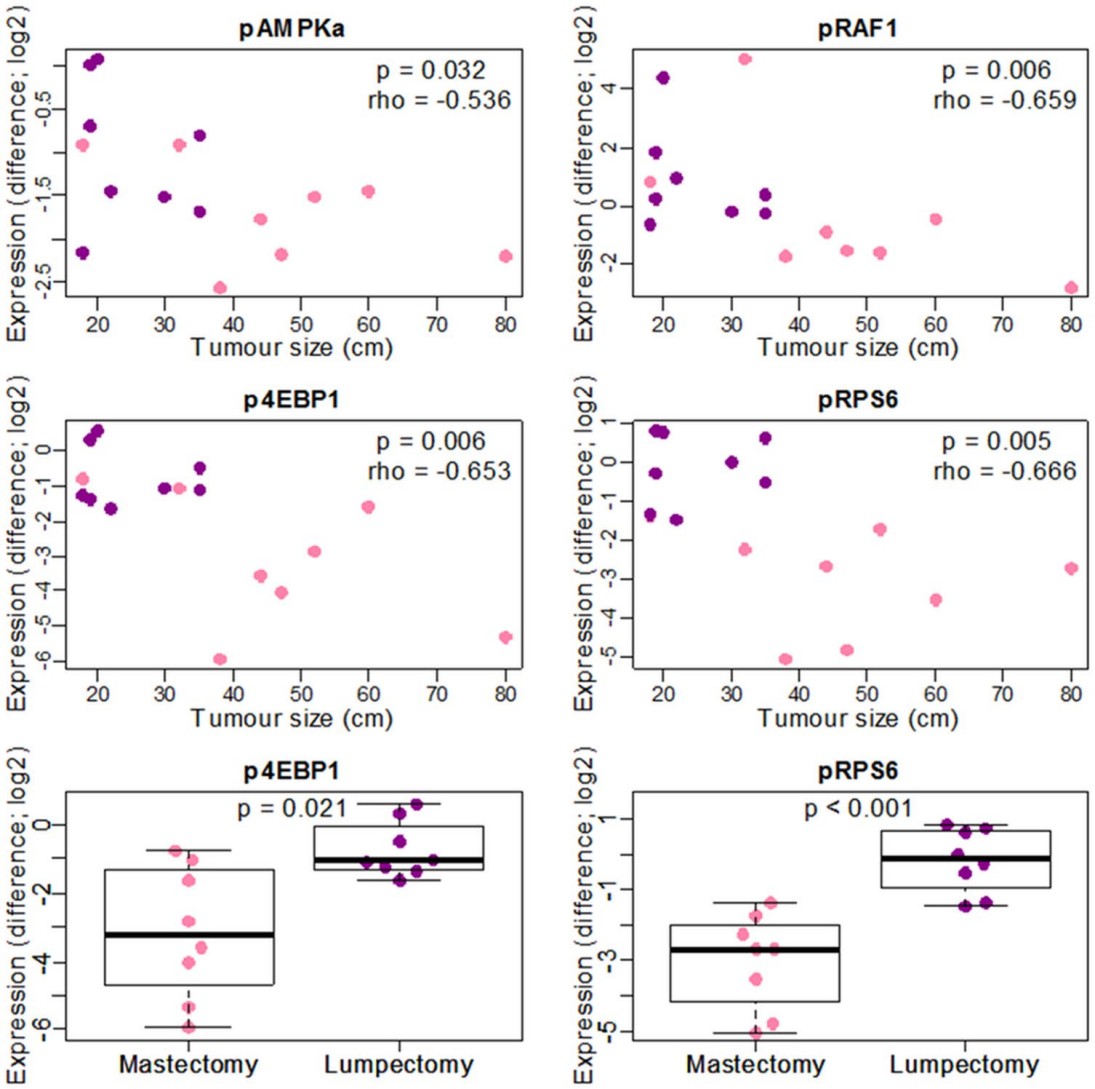
Phosphorylation immunogenicity and tumour size or type of surgery. Pink: mastectomy. Purple: lumpectomy. For tumour size, *p*-value and coefficient of correlation (*ρ*) of Spearman Correlation test are shown. For type of surgery, *p*-value of Mann–Whitney *U* test is shown. Difference = log2(Excision) − log2(Core-cut). Zero values were set as half of the lowest expression detected of the respective protein

As expected, tumours were larger in patients treated with mastectomy (median = 45.5 mm, IQR = 24.5) compared to lumpectomy (median = 21 mm, IQR = 14.75; *p* = 0.015). The difference in immunogenicity between paired core-cuts and surgical excisions was significantly correlated with tumour size for 4 markers (Fig. 4): p4EBP1 (*p* = 0.006; *ρ* = − 0.653), pAMPKa (*p* = 0.032; *ρ* = − 0.536), pRPS6 (*p* = 0.005, *ρ* = − 0.666) and pRAF1 (*p* = 0.003; *ρ* = − 0.696). The difference in expression of p4EBP1 (*p* = 0.021) and pRPS6 (*p* < 0.0001) between paired core-cuts and surgical excisions was also significant in mastectomy compared with lumpectomy (Fig. 4).

### Correlation between differences in non-phosphoproteins and phosphorylated proteins immunoreactivity

Since the difference in the expression of phosphorylated proteins between core-cuts and excision specimens may be a result of delayed fixation process, we also evaluated whether the difference between core-cuts and surgical excisions in the expression of phosphorylated and non-phosphorylated proteins were correlated. In general, most of the phosphoprotein differences were strongly correlated with one another and most of non-phosphorylated proteins were also strongly correlated with one another (Supplemental Fig. 2). The mean difference of all phosphorylated proteins was correlated with that of all non-phosphorylated proteins (*p* < 0.001; *ρ* = 0.785; Supplemental Fig. 3). The mean difference of phosphorylated proteins immunoreactivity was correlated with changes of 8/13 non-phosphorylated proteins (Supplemental Figs. 2 and 3): 4EBP1 (*p* = 0.019; *ρ* = 0.588), ERK1/2 (*p* = 0.028; *ρ* = 0.556), GSK3B (*p* < 0.001; *ρ* = 0.800), HER2 (*p* < 0.001; *ρ* = 0.841), Ki67 (*p* = 0.003; *ρ* = 0.697); MET (*p* = 0.018; *ρ* = 0.591), pan-AKT (*p* = 0.004; *ρ* = 0.697), TSC2 (*p* = 0.001; *ρ* = 0.741). These significant positive correlations were despite the mean level of some of non-phosphorylated proteins being significantly higher in surgical excisions than core-cuts and the overall mean level of the phosphorylated proteins being significantly reduced.

## Discussion

The quantification of protein expression in FFPE samples, the most frequently available tissue for analysis, is usually performed with low throughput/singleplex methods such as standard IHC. Although several advances over the last years have been described for quantification of IHC i.e. digital analysis and IF staining, IHC still has several limitations and relatively low throughput. Large-scale analyses of proteins by mass spectrometry have also been developed, but this technique requires high level of specialization for measurement and data analysis [16]. On the other hand, gene expression molecular assays have gained widespread use to allow fast and sensitive quantification of thousands of genes [17]. Recently, panels of DNA bar-coded antibodies have become available that allow rapid and simultaneous measurement of multiple proteins. The method described here applies the same end-technology currently used for RNA and DNA analysis on the NanoString nCounter platform with general high sensitivity and reproducibility [12]. Noteworthily, only pEGFR had counts below that detected for IgG antibodies (controls for non-specific binding) in all samples. These data agree with the consistent reports of very low expression of EGFR in ER positive breast cancer which our cohort was formed from exclusively [18, 19]. However, since this method is still based on antigen–antibody binding, the effect of pre-analytical variables needs to be characterized to ensure reproducibility and analytic validity before widespread use in investigations using clinical FFPE samples.

Our data show a strong correlation between standard IHC and NanoString technology for protein expression analysis providing initial support for the validity of the NanoString technique in both core-cuts and surgical excisions. We estimated that in our previous study [14] 7 h bench time was necessary to score Ki67, PgR, HER2, pAKT and pERK1/2 for 12 samples. In contrast, approximately 2 h bench time (including incubation time) was necessary to perform all the steps after antibody incubation to obtain the normalized counts for 26 proteins in 12 samples (a batch) using the new technology. While the higher cost of this new approach is likely to prevent it replacing IHC for the small number of biomarkers routinally measured in primary breast cancer, it may be cost-effective in clinical research protocols that often include the assessment of large number of biomarkers particularly phosphorylated markers [20]. Another advantage of the NanoString approach is its inclusion of within-sample housekeeping probes (such as Histone 3) that help to correct for variability in the analytical process.

The result of the PgR expression comparison between the two techniques should be interpreted with caution. In the IHC, we used an antibody that recognizes both isoforms A and B of PgR as is standard clinical practice. On the other hand, only isoform B was measured using the nCounter^®^ Vantage 3D™ Panel for FFPE, limiting our interpretation. Currently the commercial panel of Nanostring reagents does not include an antibody-probe to oestrogen receptor although this is a widely measured analyte. Given the overall encouraging data derived and reported here we have initiated a bespoke assessment of oestrogen receptor with NanoString technology.

The comparison of measurements between core-cuts and surgical excisions revealed a large number of differences that could cause major inaccuracies if not recognized. Only 2 surgical excisions showed mean phosphorylation higher than in core-cuts from the same tumours and a dramatically lower level of most of the phosphorylated proteins (8/12) immunoreactivity was detected in tumour excisions, including the loss of pERK1/2 and pAKT as previously reported [14, 21, 22]. This systematic change between core-cuts and surgical excisions is most likely to be a result of a longer time to achieve fixation in the excision samples. At room temperature and without fixation accelerators, formalin penetrates tissue at about 1 mm/h [23]. Thus core-cuts (small volume) are rapidly fixed after immersion in formalin. Conversely, formalin penetration of the larger volume specimens is a slower process, which results in greater loss of biomarker immunoreactivity. The effect of the delay fixation in larger volume specimens was confirmed directly for some markers in this study and further supported by the greater reduction of phosphorylated protein expression in mastectomies compared to lumpectomies. Although ASCO/CAP guidelines suggest samples reach fixative in less than 1 h for analysis of common biomarkers in clinical practice [4, 15], our findings support the suggestion by Ibarra *et al*. that more focus should be given on time before fixation of thinly sliced specimens rather than the time the tissue remains in formalin [24].

The extreme loss of phosphorylated protein expression can lead to difficult or even erroneous interpretation of data in clinical studies involving excision specimens. Phosphorylated proteins are often considered as biomarkers of active kinase signalling pathways and the reduction in the level of such proteins as indices of inhibition of the respective pathways. In breast cancer, the “window of opportunity” between diagnosis and surgical excision has frequently been exploited to assess the biological impact of medical treatments [25–27]. Comparisons between the routinely available diagnostic core-cuts and surgical excisions in that scenario could lead to reductions in levels of phosphoproteins being erroneously interpreted as indicating pharmacologic activity of the respective agent. Some phosphoproteins are more labile, such as pERK1/2 [21], and thus it is expected that the impact of delay in fixation will vary according the type of surgery as well as the size of tumour. Consistent with this there was a small number of phosphoproteins in the panel examined that were not significantly different in their expression between core-cuts and surgical excisions. The key message is to be aware that many phosphoproteins are grossly affected by the sample type/fixation time but it should not be assumed that a particular phospho-marker of interested is affected: it should be tested. While this loss of phosphoprotein immunoreactivity is an issue that is best avoided in clinical protocols, the inclusion of paired cores and excision samples in our study allowed us to demonstrate the similar impact of the artefact with both methods.

Although a consistently lower expression of most phosphoproteins expression was detected in excision specimens, the non-phosphorylated proteins pan-KRT, MET, 4EBP1 and ERK1/2 showed significantly high expression these samples. Given this, it was initially surprising to observe that there was a significant positive correlation between the overall difference in expression of phosphoproteins between core-cuts and surgical excisions with the expression of these non-phosphoproteins. A possible explanation of this is that the increased expression of the non-phosphoproteins may be due to a stress response, including to hypoxia [28]. For example, increased of expression of ERK1/2 may be related to its role in survival under hypoxia conditions in breast cancer cells [29]. We have observed increases in many RNA transcripts in samples with just a 20–60 min delay before fixation [30, 31]. The correlation could then be also explained by these non-phosphoproteins also being labile such that the apparently lesser degree of difference in expression between some core-cuts and surgical excisions would be found in those paired samples where delays to fixation were greatest.

The present study has some limitations. Firstly, the number of samples analysed was relatively small: it was clearly sufficient to demonstrate the compatibility of major breast cancer biomarker and of the differences between core-cuts and surgical excisions; however, larger numbers would be needed to identify any problems that might occur in some other sample types. Secondly, only 5 proteins were compared between NanoString technology and standard IHC. Thirdly, samples were collected and the original IHC data obtained in 2008–2009, in 8–9 years before NanoString analysis was performed. Antigenicity, however, is largely stable in tumour blocks as used here as opposed to stored sections [28] and the strong correlation seen between the technologies and between markers suggests this was not a significant limitation. Third, the studied sample was composed by only ER+ tumours and only one case was classified as HER2+ by IHC analysis. Therefore, cohorts of HER2+ tumours should be assessed to determine the ability of this technology to segregate HER2− and HER2+ tumours reliably. Lastly, due to the exploratory nature of the study, we did not correct for multiple comparisons involving 25 proteins and acknowledge that false-positives may have arisen. It should also be noted that NanoString technology does not take into consideration the pathologists’ assignment of the location of the positive cells as in IHC; however, we dissected away the non-invasive tissue areas prior the antibody incubation to reduce the impact of benign tissue.

In conclusion, our study provides preliminary support for the analytic validity of the new bar-coded multiplex approach to protein analysis but indicates that as with IHC caution is necessary for the analysis in surgical excisions specimens particularly of many phosphoproteins. The type of tissue specimen and type of surgery may act as confounding factors in drug discovery or development of companion diagnostic tests based on protein analysis.

## Electronic supplementary material

Below is the link to the electronic supplementary material.

Supplementary material 1 (PDF 404 KB)

Supplementary material 2 (PDF 110 KB)

## Abbreviations

IHC: Immunohistochemistry
DAB: Diaminobenzidine
IF: Immunofluorescent
FFPE: Formalin-fixed paraffin-embedded
IQR: Interquartile range
RMH: Royal Marsden Hospital
HPF’s: High-power fields
H&E: Haematoxylin and eosin
ERCC: External RNA controls consortium
