# Supplementary material for: Comparison of protein expression between formalin-fixed core-cut biopsies and surgical excision specimens using a novel multiplex approach
Source: Breast Cancer Res Treat. 2019 Feb 22;175(2):317–26. doi: 10.1007/s10549-019-05163-6 (PMC6533418; doi:10.1007/s10549-019-05163-6)
Supplement: Supplementary file 1 — Supplementary material 1 (PDF 404 KB) [file 10549_2019_5163_MOESM1_ESM.pdf]

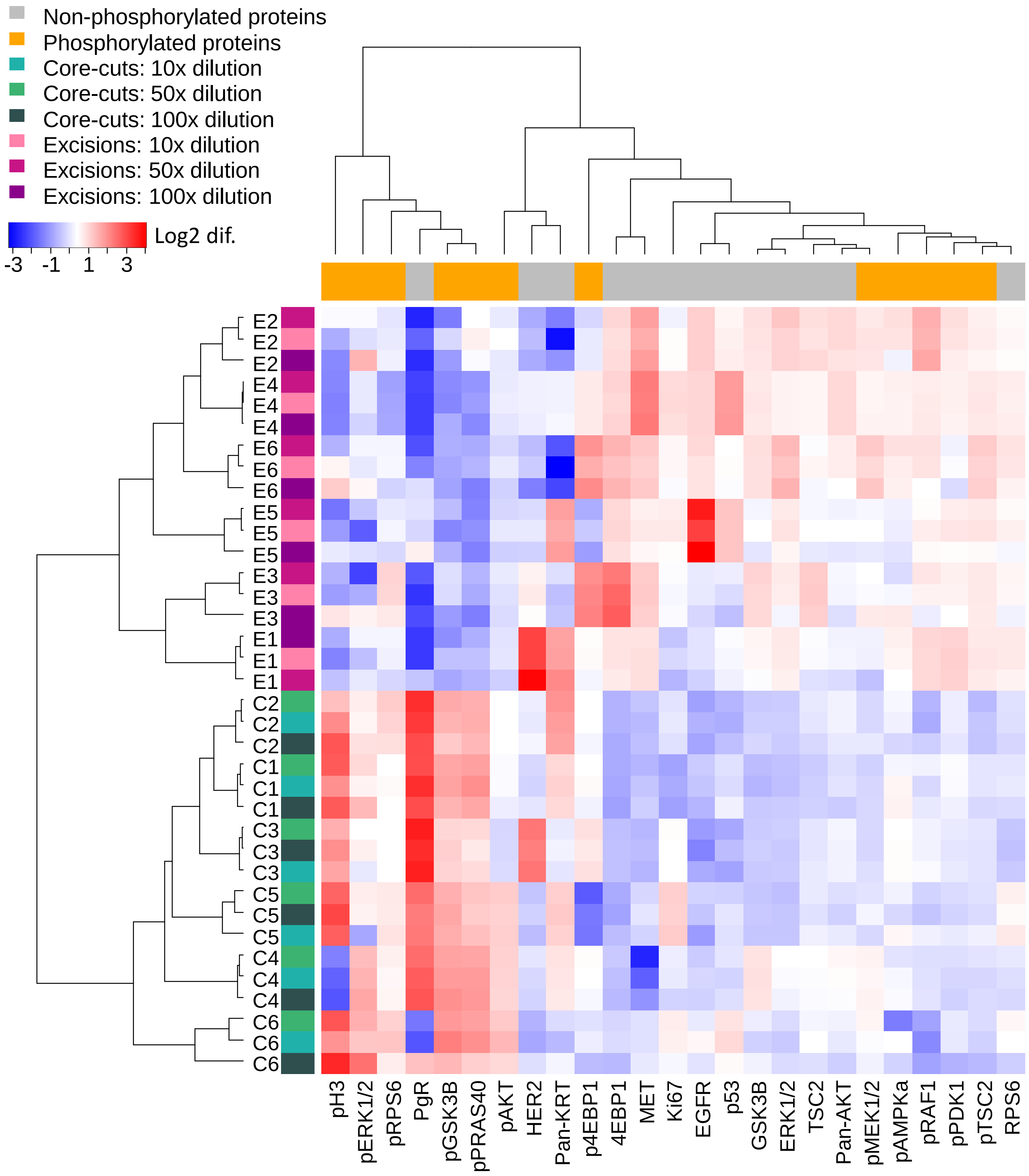

Supplementary figure 1. Heatmap based on Spearman Correlation test. Columns values were scaled by log2 difference in relation to the mean of each protein. Grey: non-phosphorylated proteins; orange: phosphorylated proteins; Pink/Purple: Core-cuts; Green: Mastectomy; C: core-cuts; E: surgical excisions. In sample bar legends, 10x dilution of cleaved tags is shown as light colours, 50x as medium colours and 100x as dark colours.

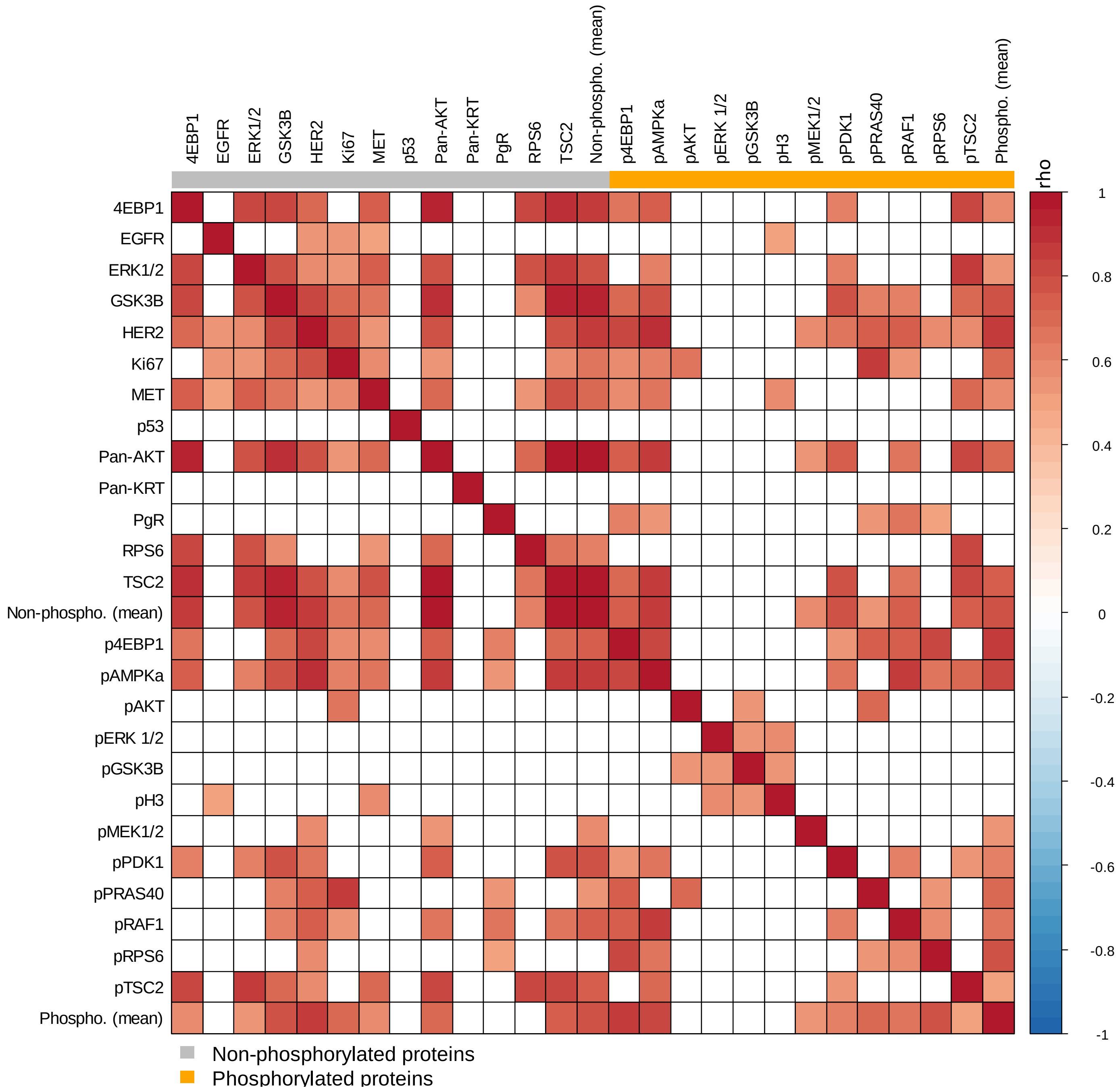

Supplementary figure 2. Correlation matrix of difference of 25 proteins between core-cuts and surgical excisions. Red and blue bar: rho coefficient of spearman correlation test. Grey: non-phosphorylated proteins; Orange: phosphorylated proteins. Blank cells: non-significant correlation (p>0.05).

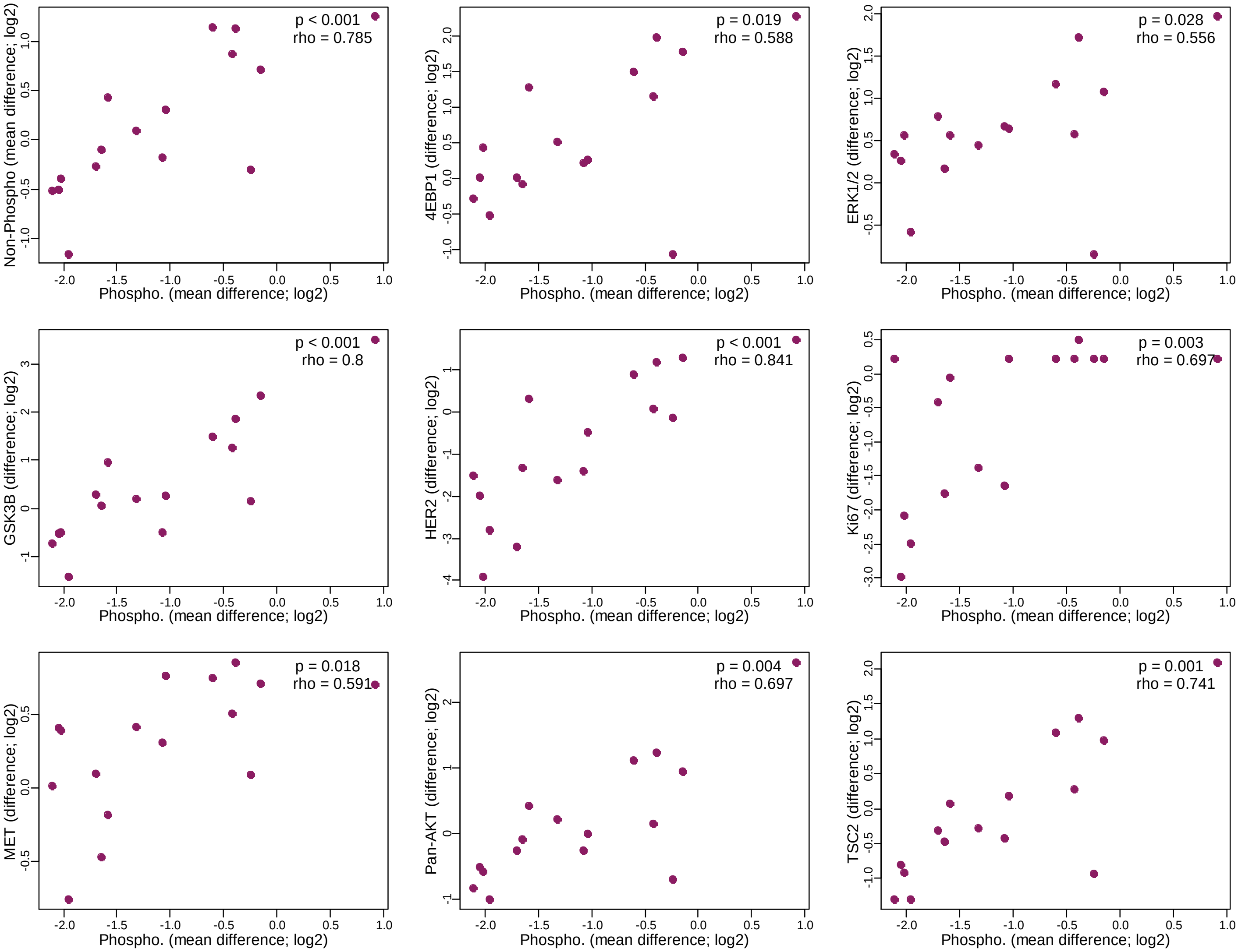

Supplementary figure 3. Correlation between the difference of the overall mean of phosphorylated proteins and the non-phosphorylated proteins between paired core-cuts and surgical excisions. rho: coefficient of spearman correlation test.
