# Supplementary material for: Comparison of protein expression between formalin-fixed core-cut biopsies and surgical excision specimens using a novel multiplex approach
Source: Breast Cancer Res Treat. 2019 Feb 22;175(2):317–26. doi: 10.1007/s10549-019-05163-6 (PMC6533418; doi:10.1007/s10549-019-05163-6)
Supplement: Supplementary file 2 — Supplementary material 2 (PDF 110 KB) [file 10549_2019_5163_MOESM2_ESM.pdf]

Supplemental table 1. Studied proteins of nCounter® Vantage 3D™ Protein Solid Tumor Panel for FFPE

						Main pathways					
	Protein name	Symbol	Clone Name	Phosphosite	Driver protein	Wnt	TGFB	MAPK	PI3K	RAS	Cell Cycle
Targets	3-phosphoinositide-dependent protein kinase 1	PDPK1	C49H2	Ser241					+		
	Cellular tumor antigen p53	p53	DO-7	N/A	+	+		+	+		+
	Dual specificity mitogen-activated protein kinase kinase 1/2	pMEK1/2	41G9	Ser217/221	+			+	+	+	
	Epidermal growth factor receptor	EGFR	D38B1	N/A	+			+	+	+	
	Epidermal growth factor receptor	pEGFR	D7A5	Tyr1068	+			+	+	+	
	Eukaryotic translation initiation factor 4E-binding protein 1	4EBP1	53H11	N/A					+		
	Eukaryotic translation initiation factor 4E-binding protein 1	p4EBP1	236B4	Thr37/46					+		
	Glycogen synthase kinase-3 beta	GSK3B	D5C5Z	N/A		+			+		+
	Glycogen synthase kinase-3 beta	pGSK3B	D85E12	Ser9		+			+		+
	Hepatocyte growth factor receptor	MET	D1C2	N/A	+				+	+	
	Histone H3	pH3	D2C8	Ser10							+
	Keratin	Pan-KRT	C11	N/A				+			
	Mitogen-activated protein kinase 1/3	EKR1/2	137F5	N/A			+	+	+	+	
	Mitogen-activated protein kinase 1/3	pERK1/2	D13.14.4E	Thr202/Tyr204			+	+	+	+	
	Non-specific serine/threonine protein kinase	pAMPKa	40H9	Thr172					+		
	Progesterone Receptor	PgR	YR85	N/A	+						
	Proliferation marker protein Ki-67	Ki67	8D5	N/A							+
	Proline-rich AKT1 substrate 1	pPRAS40	D4D2	Thr246					+		
	RAC serine/threonine-protein kinase	Pan-AKT	C67E7	N/A	+			+	+	+	+
	RAC serine/threonine-protein kinase	pAKT	D9E	Ser473	+			+	+	+	+
	RAF proto-oncogene serine/threonine-protein kinase	pRAF1	Polyclonal	Ser259				+	+	+	
	Receptor tyrosine-protein kinase erbB-2	ERBB2*	29D8	N/A	+			+	+	+	
	S6 Ribosomal Protein	RPS6	54D2	N/A					+		
	S6 Ribosomal Protein	pRPS6	D57.2.2E	Ser235/236					+		
	Tuberin	TSC2	D93F12	N/A					+		
	Tuberin	pTSC2	5B12	Thr1462					+		
Controls	Histone H3	HIST1H3A	D1H2	N/A							
	Mouse Immunoglobulin G	IgG	MOPC-21	N/A							
	Rabbit Immunoglobulin G	IgG	DA1E	N/A							

*HER2; N/A: not applied.
